# Nationwide seroprevalence of *Mycobacterium bovis* and *Mycobacterium avium* in domestic sows and wild boars in Korea under a one health framework

**DOI:** 10.3389/fmicb.2026.1773768

**Published:** 2026-02-26

**Authors:** Seon Jae Moon, Da-Yun Bae, Yun-Chae Cho, Dae Sung Yoo, Yeonsu Oh, Ho-Seong Cho

**Affiliations:** 1 College of Veterinary Medicine and Biosafety Research Institute, Jeonbuk National University, Iksan, Republic of Korea; 2 Department of Preventive Veterinary Medicine, College of Veterinary Medicine, Chonnam National University, Gwangju, Republic of Korea; 3 College of Veterinary Medicine and Institute of Veterinary Science, Kangwon National University, Chuncheon, Republic of Korea

**Keywords:** domestic sows, *Mycobacterium avium*, *Mycobacterium bovis*, one health surveillance, seroprevalence, wild boar (*Sus scrofa*)

## Abstract

**Introduction:**

Tuberculosis caused by *Mycobacterium bovis* and infections due to nontuberculous mycobacteria, particularly the *Mycobacterium avium* complex (MAC), are increasingly recognized at the livestock–wildlife–human interface. In the Republic of Korea, bovine tuberculosis remains endemic in cattle, yet nationwide data on mycobacterial exposure in suids are lacking.

**Methods:**

Between February 2023 and November 2024, serum samples from 1,366 domestic sows and 1,168 wild boars collected across nine administrative provinces were analyzed using validated commercial ELISAs to estimate apparent seroprevalence.

**Results:**

Apparent seroprevalence of *M. bovis* was 4.54% (95% CI: 3.56%–5.78%) in domestic sows and 5.91% (95% CI: 4.69%–7.41%) in wild boars. Apparent seroprevalence of **M. avium** was 10.10% (95% CI: 8.61%–11.81%) in domestic sows and 7.71% (95% CI: 6.31%–9.38%) in wild boars. Significant provincial variation was detected only for **M. avium** in domestic sows and was driven by higher seropositivity in Gyeonggi Province, whereas no significant province-level heterogeneity was observed in wild boars.

**Discussion:**

Because ELISA-based serology reflects exposure rather than active infection, results should be interpreted with caution. In an international context, the observed seroprevalence in Korea was higher than that reported from intensive indoor production systems but lower than estimates from wildlife-rich ecosystems with established reservoir hosts. These findings indicate ongoing environmental exposure to mycobacteria in Korean suids and support the need for integrated One Health surveillance incorporating domestic pigs, wildlife, and complementary diagnostic approaches.

## 1 Introduction

Tuberculosis (TB) caused by *Mycobacterium bovis* and other members of the *Mycobacterium tuberculosis* complex (MTC) remains a persistent challenge at the livestock–wildlife–human interface. Although long-standing eradication programs have substantially reduced bovine TB in many regions, MTC continues to circulate in wildlife reservoirs and may spill over into domestic animals, including pigs. Under certain ecological conditions, pigs can function as spillover or bridge hosts within multi-host systems, thereby contributing to environmental persistence and transmission opportunities (Di Marco et al., 2012; Cano-Terriza et al., 2018). Evidence from Mediterranean ecosystems, including Spain and Italy, has demonstrated MTC infection in domestic and wild swine populations, highlighting their epidemiological relevance where livestock, wildlife, and shared environments overlap (Di Marco et al., 2012; Cano-Terriza et al., 2018; Muñoz-Mendoza et al., 2013; Iovane et al., 2020). In contrast, countries dominated by intensive indoor pig production systems typically report low prevalence, emphasizing the influence of production ecology and wildlife contact on exposure risk (Campbell et al., 2011; Pedersen et al., 2017; Sun et al., 2024).

In the Republic of Korea, bovine TB has remained endemic for more than a century, with recent outbreak continuing despite sustained national efforts. In parallel, MTC infections have been documented in suids (Ku et al., 2016; Seo et al., 2017). *M. bovis* isolated from a wild sow (*Sus scrofa*) in 2012, exhibited a genotype distinct from strains circulating in cattle and deer, suggesting an independent or previously unrecognized source of exposure (Ku et al., 2016). In the same year, *M. tuberculosis* infection was confirmed in a domesticated wild boar through PCR and immunohistochemistry (Seo et al., 2017). Although such reports are sporadic, they indicate that domestic pigs and wild boars in Korea may be exposed to MTC and could participate as bridge hosts at the livestock–wildlife interface – an issue central to One Health surveillance.

Beyond MTC, the *Mycobacterium avium* complex (MAC), comprising *M. avium* and *M. intracellulare*, presents additional challenges for veterinary and public health sectors. MAC organisms are widespread in the environment, and pigs are susceptible to infection, often developing granulomatous lesions in mesenteric lymph nodes detected at slaughter. Although infections are frequently subclinical, MAC is associated with carcass condemnation and economic loss and may carry zoonotic implications, particularly for immunocompromised individuals (Hulinova Stromerova and Faldyna, 2018). In Korea, the incidence of MAC pulmonary disease in humans has increased, and *M. avium* is now frequently isolated in clinical settings (Kwon et al., 2019). Despite these trends, MAC exposure in Korean swine remains poorly characterized, limiting understanding of potential environmental or cross-species exposure routes. Notably, MAC, especially *M. avium* subsp. *hominissuis*, has been frequently detected in slaughter pigs and linked to lymph node lesions, underscoring its relevance to herd health and the food chain (Gcebe et al., 2023).

International studies report wide variability in mycobacterial prevalence in domestic pigs and wild suids, reflecting differences in production systems, wildlife reservoirs, and diagnostic approaches. Higher prevalence has been reported in wildlife-rich ecosystems and in settings with outdoor husbandry or established maintenance hosts, whereas low prevalence is typically observed in intensive indoor systems. However, direct comparisons among studies are constrained by methodological heterogeneity, including differences in the use of serology, PCR, culture, lesion inspection, or skin testing.

Taken together, the persistence of bovine TB, the occurrence of MTC infections in suids, and the growing public health significance of NTM – including MAC – highlight the need for integrated surveillance across animal species. Yet, no nationwide serological survey of *M. bovis* or *M. avium* exposure in swine has been conducted in Korea. This knowledge gap hampers risk assessment at the livestock–wildlife–human interface.

This study therefore aimed to (i) provide the first nationwide estimates of apparent seroprevalence of *M. bovis* and *M. avium* in domestic sows and wild boars in Korea, (ii) evaluate province-level heterogeneity while avoiding inference about spatial clustering beyond the resolution of the data, and (iii) position Korean swine mycobacterial exposure within a global One Health context.

## 2 Materials and methods

### 2.1 Sample size determination and sample collection

Minimum sample sizes for domestic sows and wild boars (*Sus scrofa*) were calculated using population estimates from the 2024 Q2 Livestock Trend Survey (Statistics Korea, 2025) and the 2023 Wildlife Survey (National Institute of Biological Resources, 2023), respectively. The source population was estimated at approximately 975,000 domestic sows and 73,523 wild boars (Table 1).

**Table 1:** Estimated provincial population sizes and minimum sample sizes for domestic sows and wild boars in Korea used for nationwide serological surveillance.

Provinces	Domestic sows		Wild boars	
	Minimum sample size	Estimated population	Minimum sample size	Estimated population
Gyeonggi	171	156,105	51	3,573
Gangwon	46	42,343	231	16,157
Chungbuk	57	52,371	65	4,566
Chungnam	226	207,557	83	5,820
Jeonbuk	127	115,733	63	4,381
Jeonnam	116	105,979	149	10,418
Gyeongbuk	140	127,697	262	18,246
Gyeongnam	133	121,241	145	10,142
Jeju	50	45,974	3	221
Total	1,066	975,000	1,052	73,523

Sample size calculations were performed in R version 4.3.2 (R Studio 2023.09.01), assuming an expected prevalence (P) of 0.5, a 95% confidence level (*Z* = 1.96), and a margin of error (d) of 0.03. A stricter margin of error than the conventional 0.05 was selected to increase the minimum required sample size and thereby improve the precision of national prevalence estimates. At the study design stage, diagnostic sensitivity and specificity were not incorporated into the sample size formula, as reliable field-validated estimates for the commercial ELISA assays in domestic pigs and wild boars under low-prevalence conditions are currently limited.

An initial sample size assuming an infinite population was calculated as:


$$n_{0}=\frac{[Z^{2}\times P\times (1-P)]\;}{\;d^{2}}$$

The final required sample size (*n*) for each species was then obtained using finite population correction:


$$n=\frac{n_{0}}{\left[1+(n_{0}-1)/N\right]}$$

where *N* denotes the estimated source population size. Under these assumptions, the minimum required sample size was 1,066 domestic sows and 1,052 wild boars. Final target sample sizes were proportionally allocated across nine administrative provinces according to regional population distributions (Table 1).

Between February 2023 and November 2024, serum samples were obtained from 1,366 domestic sows and 1,168 wild boars across nine provinces. For domestic sows, approximately 5 mL of blood was collected via jugular venipuncture into serum separator tubes (Ampulab Serum Separation Gel Tube; Soyagreentec, Seoul, Korea). Samples were transported on ice to the Swine Disease Laboratory at the College of Veterinary Medicine, Jeonbuk National University within a maximum of 24 h after collection, centrifuged at 3,000 × *g* for 10 min, and stored at −20 °C until analysis. Domestic sow samples were collected from breeding herds maintained under intensive indoor production systems. Wild boar samples were obtained post-mortem from animals harvested through routine population management and regulated hunting programs coordinated by local authorities. Blood samples were collected immediately after harvest and processed using the same laboratory procedures as domestic sow samples.

### 2.2 ELISA-based serological detection

All sera were tested for antibodies to *M. bovis* and *M. avium* using commercial ELISA kits (ID Screen® Porcine Tuberculosis Indirect and ID Screen® *Mycobacterium avium* Indirect Multi-species; Innovative Diagnostics, Grabels, France), following the manufacturer’s instructions. Samples were diluted 1:100 in dilution buffer, and optical densities (OD) were measured at 450 nm.

The sample-to-positive (S/P) ratios were calculated as:


$$S/P=\frac{\left(\mathrm{OD}\_\text{sample}-\mathrm{OD}\_\text{negative control}\right)}{\left(\mathrm{OD}\_\text{positive control}-\mathrm{OD}\_\text{negative control}\right)}\times 100$$

Following the manufacturer’s instructions, S/P ratios ≥ 40 (for *M. bovis*) or ≥ 50 (for *M. avium*) were interpreted as positive. Positive and negative control sera supplied with the kit were included on each plate to validate assay performance according to the manufacturer’s quality criteria (OD ratio > 3.0 between controls).

### 2.3 Statistical analyses

Apparent seroprevalence was calculated as the proportion of ELISA-positive samples among those tested. Exact two-sided 95% confidence intervals (CIs) were computed using the Clopper–Pearson method (binom.test in R), which is conservative and suitable for low-prevalence settings.

Provincial differences were assessed using Fisher’s exact test when expected cell counts were sparse (<5), and Pearson’s chi-square test when assumptions were met. Specifically, Fisher’s exact test was applied for *M. bovis* in sows and for both antigens in wild boars, whereas Pearson’s chi-square test was used for *M. avium* in sows. When global testing indicated significance (*α* = 0.05, two-sided), post-hoc cellwise diagnostics were performed using standardized Pearson residuals; |z| ≥ 1.96 was considered noteworthy. *p*-values were adjusted across provinces (*m* = 9) using Holm’s method.

Samples with missing or indeterminate ELISA results were excluded under complete-case analysis; however, no samples were excluded for this reason (0 of 2,534 samples). A farm was classified as seropositive if ≥1 sampled sow from that farm tested ELISA-positive. As supplementary summaries, province-specific prevalence ratios with 95% CIs were calculated. True prevalence was not estimated because robust sensitivity and specificity values for these ELISAs in domestic pigs and wild boars under low-prevalence field conditions were not consistently available.

## 3 Results

### 3.1 Apparent seroprevalence of *Mycobacterium bovis* antibodies

**Figure 1:**
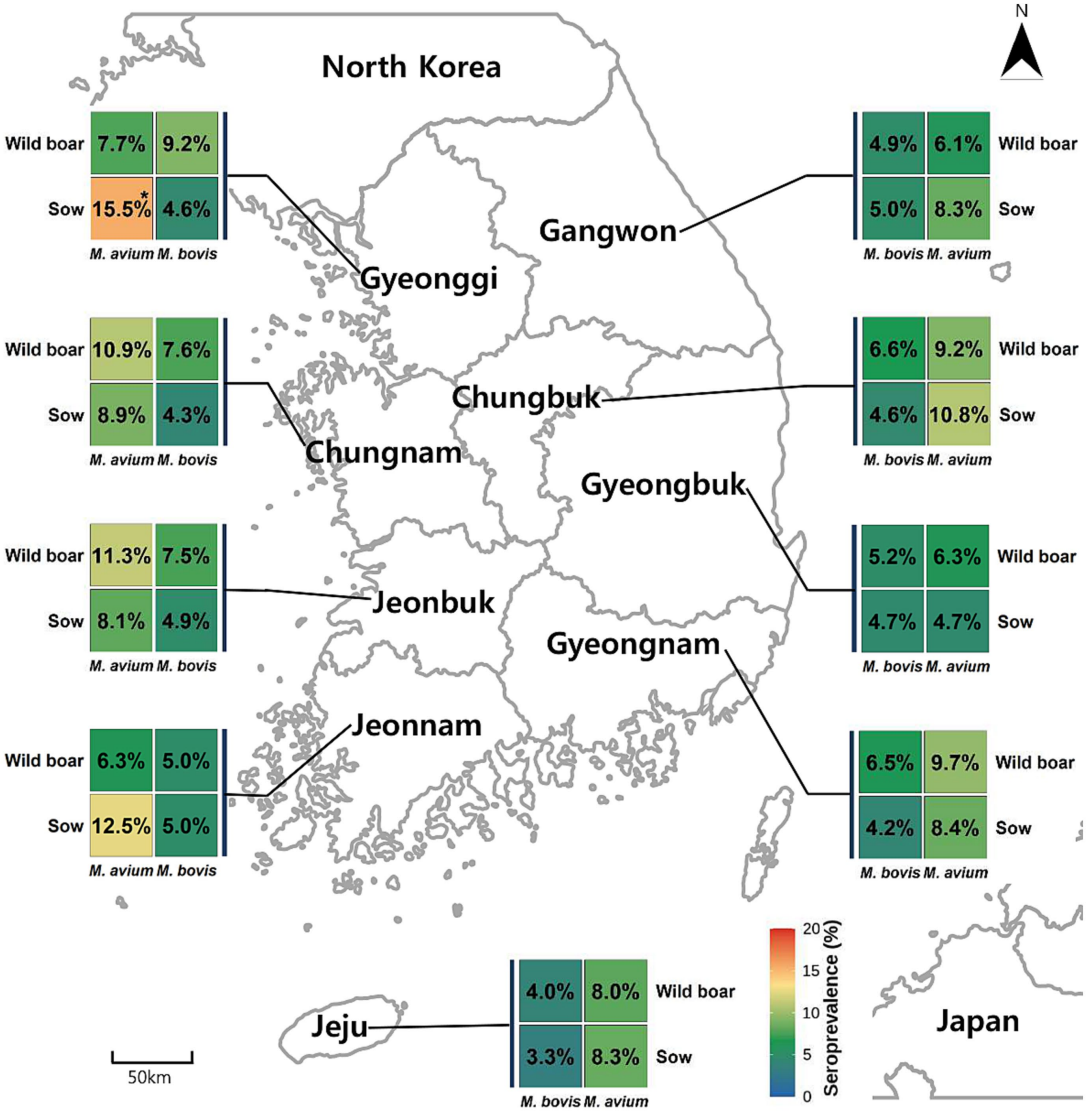
Provincial seroprevalence of *Mycobacterium bovis* and *Mycobacterium avium* antibodies in domestic sows and wild boars across Korea (2023.02–2024.11). Choropleth maps illustrate the apparent seroprevalence (%) of *M. bovis* and *M. avium* in **(A)** domestic sows (*n* = 1,366) and **(B)** wild boars (*n* = 1,168) across nine administrative provinces of Korea, based on commercial ELISA results (ID Screen® Porcine Tuberculosis Indirect; ID Screen® *M. avium* Indirect Multi-species). Provincial differences were evaluated using Pearson’s chi-square test or Fisher’s exact test, as appropriate. Significant regional variation was observed only for *M. avium* seropositivity in domestic sows, with higher-than-expected prevalence in the Gyeonggi region (Holm-adjusted *p* < 0.01). Provinces with limited wild boar sample sizes (e.g., Jeju) should be interpreted with caution.

Among 1,366 sow sera, 62 tested positive for *M. bovis* antibodies, yielding an apparent seroprevalence of 4.54% (95% CI: 3.56%–5.78%) (Table 2). Provincial seroprevalence values ranged from 3.33% to 5.00%, with no statistically significant differences detected among provinces (Fisher’s exact test, *p* > 0.05). The provincial distribution is shown in Figure 1.

**Table 2:** Apparent seroprevalence of *Mycobacterium bovis* in domestic sows and wild boars in Korea.

Region (Province)	No. of Positive sows / tested	Apparent prevalence (%) (95% CI)	No. of positive pig farms / tested	Apparent prevalence (%) (95% CI)	No. of positive wild boars / tested	Apparent prevalence (%) (95% CI)
Gyeonggi	14/303	4.62 (2.77–7.61)	11/92	11.96 (6.81–20.15)	6/65	9.23 (4.30–18.71)
Gangwon	3/60	5.00 (1.71–13.70)	2/18	11.11 (3.10–32.80)	12/245	4.90 (2.82–8.36)
Chungbuk	3/65	4.62 (1.58–12.71)	2/19	10.53 (2.94–31.39)	5/76	6.58 (2.84–14.49)
Chungnam	12/280	4.29 (2.47–7.34)	9/78	11.54 (6.19–20.50)	7/92	7.61 (3.73–14.88)
Jeonbuk	9/185	4.86 (2.58–8.99)	6/53	11.32 (5.28–22.34)	6/80	7.50 (3.48–15.41)
Jeonnam	6/120	5.00 (2.31–10.48)	4/35	11.43 (4.54–25.95)	8/160	5.00 (2.56–9.56)
Gyeongbuk	7/150	4.67 (2.28–9.32)	5/46	10.87 (4.74–23.06)	14/270	5.19 (3.11–8.51)
Gyeongnam	6/143	4.20 (1.94–8.85)	5/45	11.11 (4.87–23.63)	10/155	6.45 (3.54–11.47)
Jeju	2/60	3.33 (0.92–11.36)	2/20	10.00 (2.79–30.10)	1/25	4.00 (0.71–19.54)
Total	62/1,366	4.54 (3.56–5.78)	46/406	11.33 (8.62–14.77)	69/1,168	5.91 (4.69–7.41)

Apparent seroprevalence was calculated as the proportion of ELISA-positive samples among those tested.

Exact two-sided 95% confidence intervals (CIs) were estimated using the Clopper–Pearson method.

Provincial differences were assessed using Fisher’s exact test. No significant differences among provinces were detected (*p* > 0.05).

Of the 1,168 wild boar samples analyzed, 69 were positive for *M. bovis* antibodies, resulting in an apparent seroprevalence of 5.91% (95% CI: 4.69%–7.41%) (Table 2). Provincial estimates ranged from 4.00% (Jeju) to 9.23% (Gyeonggi), but no statistically significant provincial differences were observed (Fisher’s exact test, *p* > 0.05).

### 3.2 Apparent seroprevalence of *Mycobacterium avium* antibodies

A total of 138 of 1,366 sows were seropositive for *M. avium*, corresponding to an apparent seroprevalence of 10.10% (95% CI: 8.61%–11.81%) (Table 3). Chi-square testing revealed significant provincial heterogeneity (*χ*^2^ = 18.3, df = 8, Holm-adjusted *p* < 0.05). The Gyeonggi Province exhibited significantly higher seropositivity (15.51%), as supported by standardized residual diagnostics (Holm-adjusted *p* = 0.007).

**Table 3:** Apparent seroprevalence of *Mycobacterium avium* in domestic sows and wild boars in Korea.

Region (Province)	No. of positive Sows / Tested	Apparent prevalence (%) (95% CI)	No. of positive pig farms / tested	Apparent prevalence (%) (95% CI)	No. of positive wild boars / tested	Apparent prevalence (%) (95% CI)
Gyeonggi	47/303	15.51* (11.87–20.02)	24/92	26.09 (18.23–35.86)	5/65	7.69 (3.33–16.78)
Gangwon	5/60	8.33 (3.61–18.07)	4/18	22.22 (9.00–45.23)	15/245	6.12 (3.75–9.85)
Chungbuk	7/65	10.77 (5.32–20.06)	4/19	21.05 (8.55–42.42)	7/76	9.21 (4.53–17.81)
Chungnam	25/280	8.93 (6.12–12.85)	14/78	17.95 (11.01–27.83)	10/92	10.87 (6.01–18.86)
Jeonbuk	15/185	8.11 (4.97–12.95)	8/53	15.09 (7.84–26.90)	9/80	11.25 (6.03–20.02)
Jeonnam	15/120	12.50 (7.72–19.60)	6/35	17.14 (8.09–32.66)	10/160	6.25 (3.43–11.12)
Gyeongbuk	7/150	4.67 (2.28–9.32)	7/46	15.22 (7.61–28.35)	17/270	6.30 (3.97–9.85)
Gyeongnam	12/143	8.39 (4.87–14.09)	8/45	17.78 (9.16–31.83)	15/155	9.68 (5.95–15.35)
Jeju	5/60	8.33 (3.61–18.07)	3/20	15.00 (5.23–36.03)	2/25	8.00 (2.22–24.97)
Total	138/1,366	10.10 (8.61–11.81)	78/406	19.21 (15.71–23.25)	90/1,168	7.71 (6.31–9.38)

Apparent seroprevalence was calculated as the proportion of ELISA-positive samples among those tested.

Exact two-sided 95% confidence intervals (CIs) were estimated using the Clopper–Pearson method.

Provincial differences were evaluated using Pearson’s chi-square test for sows and Fisher’s exact test for wild boars.

*Indicates statistically significant difference after Holm adjustment (*p < 0.05).

In wild boars, 90 of 1,168 samples were seropositive for *M. avium*, resulting in 7.71% (95% CI: 6.31–9.38%) (Table 3). Provincial prevalence ranged from 6.12% (Gangwon) to 11.25% (Jeonbuk), with no statistically significant provincial variation (Fisher’s exact test, *p* > 0.05).

### 3.3 Contextual comparison with global prevalence data

For broader epidemiological context, Table 4 compares prevalence estimates from the present study with reported swine tuberculosis prevalence worldwide, derived using diverse diagnostic methods including ELISA, PCR, bacteriology, and skin testing. Seroprevalence estimates for Korean sows and wild boars fall within the lower-to-middle range of reported international values; however, comparisons should be interpreted cautiously due to methodological heterogeneity across studies.

**Table 4:** Global prevalence estimates and detection methods for swine tuberculosis in domestic pigs and wild suids, including results from the present study (Korea).

Country	Year	Sampling time	No. positives	No. tested	Prevalence	Detection method	Species
Korea (present study)	2024	2023.02–2024.11	62 (sows); 69 (wild boars)	1,366 (sows); 1,168 (wild boars)	0.045/0.059	ELISA	Pigs / Wild boars
Africa							
Burkina Faso (Sanou et al., 2021)	2021	2017.08–2017.12	8	2,430	0.003	Bacteriology	Pigs
Egypt (Mohamed et al., 2009)	2009	2004.04–2005.10	30	745	0.040	PCR	Pigs
Ethiopia (Arega et al., 2013)	2013	2011.03–2011.09	12	841	0.014	Bacteriology	Pigs
Ethiopia (Demissie et al., 2020)	2020	2016.09–2017.12	10	329	0.030	Tuberculin skin test	Pigs
Morocco (El Mrini et al., 2016)	2016	2009–2011	6	43	0.140	PCR	Wild boars
South Africa (Roos et al., 2018)	2018	2013–2015	64	170	0.376	ELISA	Warthogs
South Africa (Neiffer et al., 2021)	2021	1999.05–2016.08	40	100	0.400	ELISA	Warthogs
South Africa (Mareledwane et al., 2024)	2024	Unclear	0	90	0	PCR	Pigs
Uganda (Muwonge et al., 2010)	2010	2008.09–2009.02	32	997	0.032	Bacteriology	Pigs
Asia							
China (Sun et al., 2024)	2024	2021.02–2021.06	3	1,379	0.002	ELISA	Pigs
India (Palanivel et al., 2011)	2011	2008.05–2010.02	9	108	0.083	Bacteriology	Pigs
India (Gupta et al., 2021)	2021	2017.04–2020.02	3	42	0.071	Bacteriology	Pigs
Malaysia (Lekko et al., 2021)	2021	Unclear	5	30	0.167	ELISA	Wild boars
Malaysia (Lekko et al., 2021)	2021	2019.04–2020.08	9	30	0.300	PCR	Wild boars
Europe							
France (Richomme et al., 2019)	2019	2014–2016	8	495	0.016	Bacteriology	Wild boars
Italy (Di Marco et al., 2012)	2012	2009	4	119	0.034	PCR	Pigs
Italy (Amato et al., 2017)	2017	2013	24	299	0.080	PCR	Pigs
Italy (Iovane et al., 2020)	2020	2016–2017	46	434	0.106	ELISA	Wild boars
Poland (Orłowska et al., 2020)	2020	2011–2017	21	55	0.382	PCR	Wild boars
Poland (Welz et al., 2023)	2023	2013–2020	46	104	0.442	PCR	Wild boars
Portugal (Santos et al., 2009)	2009	2005–2007	18	162	0.111	Bacteriology	Wild boars
Portugal (Madeira et al., 2017)	2017	2011.06.01–2014.05.31	191	2,191	0.087	PCR	Wild boars
Portugal (Santos et al., 2018)	2018	2006–2013	16	678	0.024	ELISA	Wild boars
Portugal (Cardoso et al., 2024)	2024	Unclear	13	211	0.062	PCR	Wild boars
Slovenia (Pate et al., 2024)	2024	2016–2017	0	676	0.000	Bacteriology	Wild boars
		2018–2019	0	132	0.000	ELISA	
Spain (Muñoz-Mendoza et al., 2013)	2013	2008–2012	33	1,275	0.026	PCR	Wild boars
			22	1,057	0.021	ELISA	
Spain (Cano-Manuel et al., 2014)	2014	2002.10–2003.2, 2003.10–2004.2, 2004.10–2005.2, 2005.10–2006.2, 2006.10–2007.2, 2008.10–2009.2, 2009.10–2010.2	280	1,102	0.254	PCR	Wild boars
Spain (Mentaberre et al., 2014)	2014	2004–2012	103	745	0.138	PCR	Wild boars
Spain (Gortázar et al., 2017)	2017	2011–2015	329	7,676	0.042	PCR	Wild boars
Spain (Cano-Terriza et al., 2018)	2018	2015–2017	82	3,622	0.023	ELISA	Pigs
Spain (Ciaravino et al., 2021)	2020	2015.09–2016.03, 2016.09–2017.03, 2017.09–2018.03, 2018.09–2019.03, 2019.09–2020.03	17	278	0.061	PCR	Wild boars
Spain (Varela-Castro et al., 2020)	2020	2010–2016	326	1902	0.171	ELISA	Wild boars
Spain (Varela-Castro et al., 2021)	2021	2010–2019	10	894	0.011	PCR	Wild boars
Switzerland (Ghielmetti et al., 2021)	2021	2017–2018	5	176	0.028	PCR	Wild boars
North America							
U.S. (Campbell et al., 2011)	2011	2010.06–2010.09	0	98	0.000	Histopathology, Bacteriology	Wild boars
U.S. (Pedersen et al., 2017)	2017	2013.10.01–2014.09.30	1	2,725	0.000	ELISA	Wild boars
South America							
Brazil (Maciel et al., 2018)	2017	Unclear	25	80	0.313	PCR	Wild boars
Argentina (Barandiaran et al., 2024)	2024	Unclear	34	311	0.109	PCR	Wild boars
New Zealand (Nugent et al., 2012)	2011	1996–2003, 2003–2007	293	785	0.373	Bacteriology	Wild boars

Prevalence estimates were derived using different diagnostic approaches, including serology, PCR, bacteriology, and skin testing, and should be interpreted in the context of methodological heterogeneity across studies. Data for countries other than Korea were compiled from Zheng et al. (2024).

## 4 Discussion

This study provides the first nationwide serological assessment of exposure to *M. bovis* and *M. avium* in domestic sows and wild boars in Korea. By collecting samples across nine administrative provinces and applying validated ELISA assays, we establish baseline data that contribute to understanding mycobacterial exposure at the livestock–wildlife interface and provide a reference point for future surveillance efforts.

The apparent seroprevalence of *M. bovis* observed in domestic sows (4.54%) exceeds levels reported from several regions characterized by intensive indoor or semi-intensive pig production systems, including southern Spain (Cano-Terriza et al., 2018). Similarly, the seroprevalence detected in wild boars (5.91%) was higher than estimates reported from Portugal (Santos et al., 2018), the United States (Pedersen et al., 2017), Iberian Atlantic Spain (Muñoz-Mendoza et al., 2013), and Slovenia (Pate et al., 2024). In contrast, the Korean estimates were lower than those reported from multi-host systems in Campania, Italy (Iovane et al., 2020), northern Spain (Varela-Castro et al., 2020), and Malaysia (Lekko et al., 2021), where wildlife–livestock interfaces are known to support sustained transmission. Substantially, higher seroprevalence has been documented in African ecosystems, where warthogs (*Phacochoerus africanus*) function as maintenance hosts, with reported values reaching 37.6%–40.0% in South Africa (Roos et al., 2018; Neiffer et al., 2021). Collectively, these comparisons suggest that Korea occupies an intermediate epidemiological position between intensive production systems with limited wildlife contact and ecosystems characterized by persistent wildlife reservoirs. Nevertheless, such comparisons should be interpreted cautiously, as reported prevalence is strongly influenced by host populations, ecological context, and diagnostic methodology.

When placed in a broader international context, detection frequencies for *M. bovis* in suids have generally been low but variable across regions. For example, individual seroprevalence in Gansu Province, China, was reported at 0.22% (Sun et al., 2024), whereas higher detection rates have been described in Mediterranean settings, including 3.4% of slaughtered pigs in Sicily, Italy (Di Marco et al., 2012), and 2.3% seroprevalence among Iberian pigs in southern Spain (Cano-Terriza et al., 2018). For the MAC, reported frequencies likewise vary, ranging from 2.4% in slaughtered pigs in Egypt (Mohamed et al., 2009) to 4.6% in wild boars from Spain’s Iberian Atlantic region (Muñoz-Mendoza et al., 2013) and 1.9% in wild boars from southern Spain (García-Jiménez et al., 2015). Relative to these reports, the apparent seroprevalence observed in Korean domestic sows and wild boars does not indicate unusually high exposure but is consistent with measurable, low-to-moderate exposure levels.

The seroprevalence profile observed in Korea likely reflects the predominance of intensive indoor pig production systems, which limit direct contact between domestic pigs and wildlife, combined with potential environmental exposure arising from other reservoirs. Bovine tuberculosis continues to be reported in Korean cattle herds (Animal and Plant Quarantine Agency, 2025; Yoo et al., 2025), and prior documentation of *M. bovis* infection in a wild sow (Ku et al., 2016) and *M. tuberculosis* detection in a wild boar (Seo et al., 2017) supports the plausibility of multi-host exposure pathways. However, serological data alone cannot identify sources of infection or infer transmission routes, and the present findings should therefore be interpreted as evidence of exposure rather than active transmission.

Exposure to *M. avium* was more common than exposure to *M. bovis* in both domestic sows and wild boars, with apparent seroprevalence reaching 10.10% and 7.71%, respectively. Direct quantitative comparisons with previous studies are constrained by methodological heterogeneity, as many investigations of MAC in pigs and wild suids rely on culture or molecular detection rather than serology. Nonetheless, international studies demonstrate considerable variability in MAC detection in slaughter pigs and wild boars (Muñoz-Mendoza et al., 2013; Mohamed et al., 2009; García-Jiménez et al., 2015). The veterinary and public health relevance of MAC infections has been increasingly recognized, given their association with carcass condemnation in pigs and the rising incidence of MAC pulmonary disease in humans, including in Korea (Hulinova Stromerova and Faldyna, 2018; Kwon et al., 2019).

In the present study, significant regional variation in *M. avium* exposure was observed only among domestic sows, with the highest apparent seroprevalence detected in the Gyeonggi region. Studies addressing MAC exposure in domestic pigs or wild boars in Korea and East Asia remain extremely limited, underscoring the lack of regional baseline data. This finding should be interpreted cautiously, as statistical significance was driven by a single province. Gyeonggi Province encompasses the highest density of large-scale swine operations and complex peri-urban interfaces, suggesting that environmental factors such as water systems, bedding materials, or bioaerosols may contribute to elevated exposure risk (Hulinova Stromerova and Faldyna, 2018; Kwon et al., 2019). In contrast, wild boars exhibited no significant regional variation in MAC seroprevalence, consistent with diffuse, low-level environmental exposure rather than localized exposure foci, as reported in European wildlife studies (Muñoz-Mendoza et al., 2013; Richomme et al., 2019; Ghielmetti et al., 2021).

The concurrent detection of antibodies to both MTC and MAC in domestic sows and wild boars highlights several One Health considerations. Environmental and indirect transmission pathways may be sufficient to sustain low-level endemic exposure even in the absence of widespread outdoor pig farming. Korea’s high baseline prevalence of human tuberculosis and the increasing incidence of MAC pulmonary disease may further contribute to shared peri-urban exposure environments (Kwon et al., 2019; Yoo et al., 2025). In addition, other wildlife or meso-mammalian species, including rodents and small carnivores, may participate in local transmission networks, analogous to the role of brushtail possums in New Zealand’s bovine tuberculosis system (Nugent et al., 2012; Nugent et al., 2015). Evidence of mycobacterial infections in Korean companion animals and wildlife—including multidrug-resistant *M. bovis* isolated from a dog (Cho et al., 2022), *M. avium* infections in birds (Lee et al., 2010), and disseminated MAC infection in a captive tiger (Cho et al., 2006)—further underscores the relevance of integrated, multi-species surveillance.

Several methodological considerations warrant acknowledgment. ELISA-based assays are well suited for large-scale screening and have been widely applied in livestock and wildlife surveillance (Santos et al., 2018; Richomme et al., 2019; Cardoso et al., 2024); however, they primarily reflect prior exposure rather than active infection and may under-detect early or low-antibody responses. Accordingly, the apparent seroprevalence reported here should be interpreted as indicative of exposure rather than infection status. Reliable estimation of true prevalence would require robust, species- and context-specific diagnostic performance parameters that are currently unavailable for ELISA-based assays in suids. Complementary diagnostic approaches, including PCR, bacterial culture, interferon-gamma release assays, and histopathology, would improve epidemiological resolution (Mohamed et al., 2009; Cardoso et al., 2024).

The absence of demographic information for wild boars, such as age and sex, precluded assessment of risk factors known to influence infection dynamics in other settings (Muwonge et al., 2010; Arega et al., 2013; Demissie et al., 2020; Sanou et al., 2021). Similarly, individual- or farm-level risk factor analyses were not possible due to limited metadata on herd characteristics and management practices. The lack of spatial analysis further restricted identification of potential geographic clusters or transmission hotspots. Future studies integrating structured metadata, environmental variables, and molecular typing approaches – including spoligotyping, variable-number tandem repeat analysis, or whole-genome sequencing – would be instrumental in clarifying transmission pathways across hosts, as demonstrated in European multi-host systems (Amato et al., 2017; Madeira et al., 2017; Orłowska et al., 2020; Ciaravino et al., 2021; Welz et al., 2023).

Although tuberculosis is designated as a “Contagious Animal Disease Type II” under Korean legislation (Yoo et al., 2025), pigs are not currently included in routine national surveillance programs. Given the long production lifespan of breeding sows and the expanding geographic distribution of wild boars in Korea (National Institute of Biological Resources, 2023), integrating serological monitoring of both populations into existing surveillance frameworks could strengthen early detection and risk assessment. Experience from European wildlife–livestock tuberculosis systems demonstrates the value of coordinated surveillance, ecological risk evaluation, and targeted control strategies (Santos et al., 2009; Cano-Manuel et al., 2014; Mentaberre et al., 2014; Gortázar et al., 2017). Expanded environmental sampling and molecular epidemiological investigations would further clarify transmission pathways across species and ecosystems.

In conclusion, domestic sows and wild boars in Korea exhibit measurable exposure to both MTC and MAC. The observed seroprevalence profile lies between that reported for intensive indoor production systems and that observed in outdoor or wildlife-rich ecosystems with established reservoir hosts (Di Marco et al., 2012; Cano-Terriza et al., 2018; Lekko et al., 2021; Roos et al., 2018; Neiffer et al., 2021; Santos et al., 2009; Maciel et al., 2018; Barandiaran et al., 2024). These findings support the importance of sustained One Health surveillance and integrated multi-species monitoring to better understand and mitigate mycobacterial exposure risk in Korea.

## Data Availability

The original contributions presented in the study are included in the article/supplementary material, further inquiries can be directed to the corresponding authors.
